# Blood smear imagery dataset for malaria parasite detection: A case of Tanzania

**DOI:** 10.1016/j.dib.2024.111169

**Published:** 2024-11-23

**Authors:** Beston Lufyagila, Bonny Mgawe, Anael Sam

**Affiliations:** a Department of Information and Communication Sciences and Engineering, The Nelson Mandela African Institution of Science and Technology, PO Box 447, Tengeru, Arusha, Tanzania; b Department of Computer Science & Engineering, Mbeya University of Science and Technology, PO Box 131, Mbeya, Tanzania

**Keywords:** Image annotation, Malaria, Malaria parasite, Medical diagnosis, Plasmodium

## Abstract

Malaria is a major public health issue in many regions of Africa, including Tanzania. The Tanzania Malaria National Strategic Plan (2021–2025) emphasizes on high-quality testing services availability, high coverage of timely diagnosis of malaria, and availability of innovative diagnostic systems for effective detection, treatment and control of malaria. This would be achieved by employing state of the art technologies like Machine learning. However, Machine learning requires diverse dataset to work effectively and efficiently. Therefore, this paper presents blood smear imagery dataset that can be used by researchers to develop computer vision systems for malaria parasite detection. The imagery dataset were acquired by setting up a 40X-2500X Real 4 K compound microscope with a 4k SONY IMX334 sensor camera mounted to it in five health centres of Tanga region. Blood samples taken according to normal routine of diagnosing patients in health care, were stained using Giemsa reagent and examined under microscope. Following these procedures, the study collected and annotated Thick infected blood smear images (n=1139); Thick uninfected blood smear images (n=1071); Thin uninfected blood smear images (n=270); and Thin infected blood smear images (n=1064). Furthermore, the curated dataset have been uploaded in a public Harvard data verse repository. In summary, the dataset aims to support the creation of diagnostic tools that improve malaria detection, thereby advancing health outcomes and aiding malaria control initiatives in Tanzania and other regions impacted by the disease.

Specifications Table

**Table:** 

Subject	Applied Machine Learning
Specific subject area	Utilizing computer vision for diagnosing malaria Plasmodium through blood smear images to enhance diagnostic accuracy in both clinical and research applications.
Type of data	Image
Data collection	High-resolution images of blood smears from clinical samples in Tanzania were obtained using a standard laboratory microscope. The microscope used was a 40X-2500X real 4 K compound microscope, equipped with 100X (S, oil) achromatic objective and WF10X and WF25X wide-field eyepieces. A 4k Camera with SONY IMX334 sensor, real-time output resolution of 4K/2K/1080P, and 8528 × 4808 (41 Megapixel) image resolution mounted to the microscope and connected to person computer via USB with frame rate of 1080@ 60FPS(4k) was used to capture the blood smear image. Images were acquired from blood smears prepared according to standard protocols with a medical laboratory expert, ensuring a representative sample of malaria cases. Inclusion criteria focused on samples with confirmed malaria infection and non-malaria infection, while exclusion criteria removed poorly stained or unclear slides. The captured images were saved in JPEG format and organised into four separate folders named as Thick infected, Thin infected, Thick uninfected, and Thin uninfected.
Data source location	The Nelson Mandela African Institution of Science and Technology (NM-AIST) Region: Arusha Country: Tanzania
Data accessibility	Repository name: Harvard Dataverse Data identification number: 10.7910/DVN/O2WVWA Direct URL to data: https://dataverse.harvard.edu/dataset.xhtml?persistentId=doi:10.7910/DVN/O2WVWA.
Related research article	None

## 1 Value of the Data

• The data can serve as a foundational resource for advancing experiments in automating diagnosis of malaria parasites.
• The dataset can be used by researchers as inputs to generate infinite size of datasets using advanced AI techniques, including Generative Adversarial Network (GAN).
• The dataset enhances diversity, and when combined with datasets from other sources, it can help create more robust and adaptable machine learning models for computer vision tasks.
• The dataset can help researchers who want to test their algorithms for image quality enhancement [1].
• The dataset can help machine learning researchers develop technological solutions for addressing health sector challenges.

## 2 Background

The motivation for compiling this dataset arose from the need to improve the efficiency and accuracy of malaria diagnosis through microscopy, especially in African regions with cases of the disease, such as Tanzania [2,3]. Malaria diagnosis traditionally depends on the manual microscopic examination of blood smear images by trained experts, a process that is labour-intensive, susceptible to subjectivity and human error, potentially leading to inaccurate results [4]. As interest grows in applying machine learning techniques to medical diagnostics, there is a clear need for diverse, well-annotated datasets to train and validate these models [4,5].This dataset was created in the context of developing automated tools for malaria detection in Tanzania, aligned with the Tanzania Malaria National Strategic Plan of 2021–2025 which emphasizes on high-quality testing services availability, timely malaria diagnosis coverage, and innovative diagnostic systems availability for malaria detection [3]. The theoretical motivation includes leveraging computer vision and deep learning methodologies to improve diagnostic accuracy and reduce the burden on healthcare systems. This dataset aids ongoing research by offering a public resource for developing and testing machine learning algorithms for malaria detection. It supports researchers in creating automated diagnostic tools, improving malaria diagnosis in resource-limited settings like Tanzania.

## 3 Data Description

This article presents thick and thin blood smear image datasets for malaria parasite detection, collected from Tanzanian health centers. The dataset contains 3544 images, each with a resolution of 3840 × 2160 pixels in JPEG format. Each image is accompanied by a label indicating whether the smear is infected or uninfected with malaria parasites.

In the repository, the data are organized into four separate folders: one folder contains images labeled as ***THICK_INFECTED*** (for infected thick smear images), ***THICK_UNINFECTED*** (for uninfected thick smear images), ***THIN_INFECTED*** (for infected thin smear images), and ***THIN_UNINFECTED*** (for uninfected thick smear images) uploaded in a zipped format. The folders are named to reflect their corresponding image classes: 'infected' (for images with malaria parasites) and 'uninfected' (for images with no malaria parasites).

**Fig. 1:**
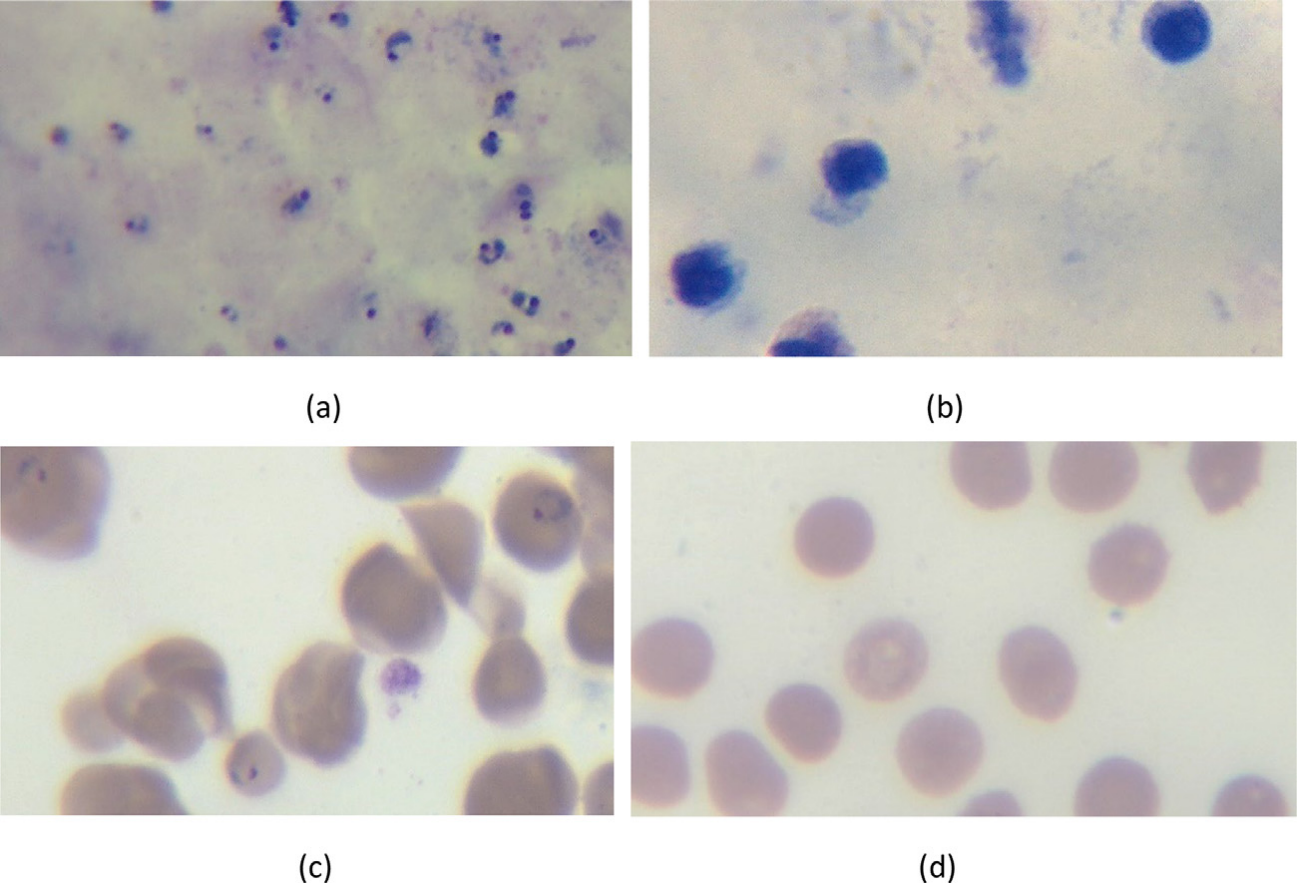
Sample images of malaria blood smears captured using a Binocular Compound Microscope (AmScope -40X-2500X LED) equipped with a 4 K digital camera with a SONY IMX334 sensor and an image resolution of 8528 × 4808 (41 megapixels) . Images were taken using 100× objective lens (magnification) under oil immersion to enhance clarity and resolution. Giemsa staining was applied to differentiate between infected and uninfected cells, as well as to distinguish leukocytes. (a) Thick infected blood smear showing multiple malaria parasites. (b) Thick uninfected blood smear showing no visible parasites but with visible leukocytes stained blue. (c) Thin infected blood smear displaying red blood cells with malaria parasites. (d) Thin uninfected blood smear showing normal red blood cells with no signs of infection.

The images were categorized into separate folders to simplify the processes of uploading, downloading, and subsequent data handling. Fig. 1 presents sample images from the dataset, showcasing both infected and uninfected images for thick blood smears, as well as infected and uninfected images for thin blood smears.

## 4 Experimental Design, Materials and Methods

### 4.1 Field data collection

Malaria thick and thin blood smear image dataset was collected by the NM-AIST located in Arusha, Northern Tanzania. A 40X-2500X Real 4 K Compound Microscope with 100X (S, oil) achromatic objective and WF10X and WF25X wide field eyepieces was used to magnify and examine the quality of a stained blood smear slide. A 4k Camera with SONY IMX334 sensor, real-time output resolution of 4K/2K/1080P, and 8528 × 4808 (41 Megapixel) image resolution mounted to the microscope and connected to a Personal computer (PC) via USB with frame rate of 1080@ 60FPS(4k) was used to capture the blood smear image. Images were acquired from blood smear slides that were prepared by fixing with methanol and staining with Giemsa reagent, following standard protocols established in collaboration with a medical laboratory expert. The data collection activity was done from May 2024 to August 2024, and involved five Health Centres (HC), which are Ngamiani, Makorora, Mikanjuni, Duga health centres, and Masiwani Hospital in Tanga region. The study collected a total of 75 blood samples, where by 40 blood samples from patients with confirmed malaria cases and 35 blood samples from health patients as summarized in Table 1. In addition, the study captured in average 50 images per patient.

**Table 1:** A summary of the blood sample size used to obtain the captured images.

Health centre	Infected Blood samples	Health blood samples	Sub- total
Ngamiani	10	10	20
Makorora	10	10	20
Mikanjuni	10	5	15
Masiwani	5	5	10
Duga	5	5	10
**Total**	**40**	**35**	**75**

### 4.2 Data preprocessing

Before being uploaded to the publicly available repository, the acquired picture data were cleaned, renamed, and annotated [6]. VisiPics V1.31 software (https://visipics.en.softonic.com/download, accessed Sep. 05, 2024) was used to delete duplicate images found during the cleaning process, while Bulk Rename Utility V3.4.4 software (https://www.bulkrenameutility.co.uk/Download.php, accessed Sep. 05, 2024) was employed for renaming the image files. Despite these efforts, a small number of near-duplicate images may still remain. Additionally, the dataset may contain distinct images that are very similar in appearance but not exact duplicates. Table 2 shows the number of malaria thick and thin smear images before and after the removal of duplicates. The curated datasets were then uploaded in the Harvard DataVerse, which is an open repository.

**Table 2:** Blood smear image types before and after duplicates and unclear images removed.

Blood smear Image type	Before duplicates and unclear images removed	After duplicates and unclear images removed
Thick infected	1165	1139
Thick uninfected	1192	1071
Thin uninfected	336	270
Thin infected	1081	1064

## Limitations

• Differences in blood smear preparation, such as smear thickness and staining intensity, created variability within the dataset. Despite efforts to standardize, these variations might not be entirely eliminated.
• The image dataset quality might not be consistence to all images due to variations in microscope settings, lighting, and staining techniques. Further image enhancement techniques can be employed to improve the quality of images before developing machine learning models.

## Ethics Statement

The study received review and approval from the Kibong'oto Infectious Diseases Hospital–Nelson Mandela African Institution of Science and Technology–Centre for Educational Development in Health, Arusha (KNCHREC). The project was carried out under Ethical Approval Certificate number: KNCHREC00018/01/2024, valid for one year. The study furthermore, received letter of approval from relevant Tanga region administrative organs—Region Commissioner (RC), District Commissioner (DC), and District Executive Director (DED) of Tanga City Council.

## Credit Author Statement

**Beston Lufyagila:** Conceptualization, Data collection, Data annotation, Writing the original draft, and Project administration. **Bonny Mgawe** and **Anael Sam:** Conceptualization and Contributed to refine research, data pre-processing, and manuscript development.

## Data Availability

DataverseThe Nelson Mandela African Institution of Science and Technology Malaria Dataset (Original data).
